# Interdisciplinary Medicine

**DOI:** 10.3390/medicina57050427

**Published:** 2021-04-28

**Authors:** Camelia Cristina Diaconu

**Affiliations:** 1 Department 5, “Carol Davila” University of Medicine and Pharmacy, 050474 Bucharest, Romania; drcameliadiaconu@gmail.com; 2 Clinical Emergency Hospital of Bucharest, 105402 Bucharest, Romania

Motto:

“*Coming together is a beginning, working together is success, keeping together is progress*”

Henry Ford.

Dear Colleagues,

Medicine of the 21st century requires multidisciplinary problem solving, for the best diagnostic and therapeutic results, to improve the healthcare of our patients.

The rapidly changing field of medicine and healthcare is increasingly adopting scientific and technological innovations, making interdisciplinary collaborations especially important. In this context, medical disciplines are becoming increasingly interlinked with other specialties and fields. A more interdisciplinary approach to the patient is needed, especially for complex patients with numerous comorbidities, most of them usually elderly and fragile. The greatest challenges to human health lie at the intersection of different medical fields. An interdisciplinary medical team is more and more necessary, with the rapid expansion of medical knowledge. Healthcare professionals need to acquire new skills regarding patient care delivery, which facilitate a better interaction between them. Healthcare is a team project and collaboration among practitioners is the key to optimal patient outcomes. Each member of the healthcare team has different and specific knowledge and skills, allowing them to contribute to a better case management. An effective interdisciplinary team may decrease the costs associated with healthcare, improve patient satisfaction, and decrease the number of medical errors and morbimortality, ultimately leading to prolonged survival of our patients and a better quality of life.

Given the importance of interdisciplinarity in the field of medicine and research, the journal *Medicina* published a very successful Special Issue on this subject, “Interdisciplinary Medicine”. The articles included in this Special Issue demonstrate that every patient is a team project. Reviews and original articles dealing with interdisciplinary medical issues, as well as articles providing an up-to-date overview of the diagnostic protocols and individualized treatments for patients with multiple comorbidities, were published. The broad international distribution of the authors, with different backgrounds, demonstrates that healthcare challenges are similar in different countries. I invite you to read the articles of “Interdisciplinary Medicine”, to understand the new directions in the field of healthcare and the challenges associated with them.

